# Graphene quantum Hall effect parallel resistance arrays

**DOI:** 10.1103/physrevb.103.075408

**Published:** 2021-02

**Authors:** Alireza R. Panna, I-Fan Hu, Mattias Kruskopf, Dinesh K. Patel, Dean G. Jarrett, Chieh-I Liu, Shamith U. Payagala, Dipanjan Saha, Albert F. Rigosi, David B. Newell, Chi-Te Liang, Randolph E. Elmquist

**Affiliations:** 1 Physical Measurement Laboratory, National Institute of Standards and Technology (NIST), Gaithersburg, Maryland 20899-8171, USA; 2 Department of Physics, National Taiwan University, Taipei 10617, Taiwan, Republic of China; 3 Physikalisch-Technische Bundesanstalt, Bundesallee 100, 38116 Braunschweig, Germany; 4 Department of Chemistry and Biochemistry, University of Maryland, College Park, Maryland 20742, USA

## Abstract

As first recognized in 2010, epitaxial graphene on SiC(0001) provides a platform for quantized Hall resistance (QHR) metrology unmatched by other two-dimensional structures and materials. Here we report graphene parallel QHR arrays, with metrologically precise quantization near 1000 Ω. These arrays have tunable carrier densities, due to uniform epitaxial growth and chemical functionalization, allowing quantization at the robust *ν* = 2 filling factor in array devices at relative precision better than 10^−8^. Broad tunability of the carrier density also enables investigation of the *ν* = 6 plateau. Optimized networks of QHR devices described in this work suppress Ohmic contact resistance error using branched contacts and avoid crossover leakage with interconnections that are superconducting for quantizing magnetic fields up to 13.5 T. Our work enables more direct scaling of resistance for quantized values in arrays of arbitrary network geometry.

## I. INTRODUCTION

The von Klitzing constant, *R*_K_ = *h*/*e*^2^, is defined in the 2019 redefinition of the SI base units as a universal reference of electrical resistance [1], and it is accessed through the integer quantum Hall effect (QHE) [2]. A single device used as a quantized Hall resistance (QHR) standard can provide only one or two highly precise resistance plateaus. Resistance scaling to artifact standards at the highest precision requires cryogenic ratio bridges, so access to a broad and useful spectrum of traceable resistance measurements is out of reach for many laboratories. This has raised the interest in constructing accessible quantum-based resistance standards using arrays of semiconductor QHE devices [3–7], and in the case of epitaxial graphene (EG), using arrays [8,9] or devices with multiple regions of opposite charge separated by sharp *p*-*n* junctions [10–12].

The magnetoresistance in EG devices is dominated by a robust plateau [13,14] at Landau level (LL) filling factor *ν* = 2, which allows precise measurements for a wide range of magnetic field *B*, source-drain current *I*, and temperature *T*. Here we describe parallel QHE arrays based on *N* interconnected EG devices. Parallel arrays of resistance (*R*_K_/*Nν*) bring practical benefits, since they provide broad areal distribution of power dissipation to increase the QHE breakdown current. This allows precise measurements with non-cryogenic measurement electronics, which are limited by current sensitivity for higher-resistance, lower-current QHR standards.

**FIG. 1.**
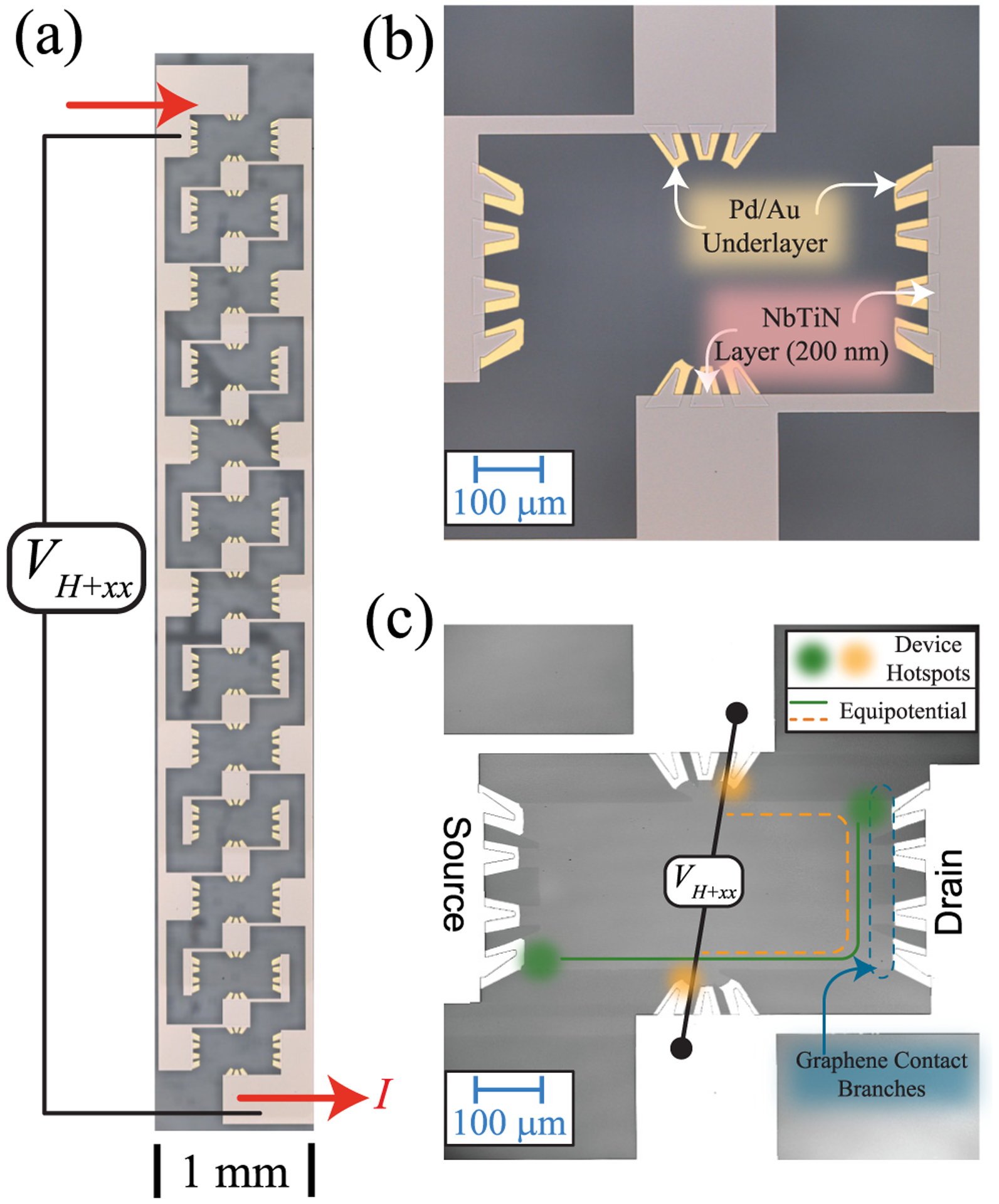
Device layout and features in a quantizing magnetic field. (a) Each of 13 array elements is 0.4 mm × 0.2 mm monolayer EG and has multiple contact branches. (b) Single array element contact layout, with a Pd/Au underlayer that provides EG surface adhesion and ensures a normal metal interface with NbTiN superconductor. (c) With strong quantization, boundaries of high and low electrochemical potential surround the EG, beginning at the two hotspots created by the power dissipation at the source and drain. These are labeled equipotential, although small changes may occur where current enters or leaves the device at intermediate contacts. The positions of hotspots depend on the orientation of *B*. In one field direction *B*+ (magnetic vector pointing into the page; equipotential boundary shown by the green line), the Hall voltage is nearly perpendicular to the source-drain current vector. This voltage is labeled *V*_*H*+*xx*_ to account for *R*_*xx*_ contributions due to the small diagonal offset of the voltage probes, as described in the text. The opposite field direction (*B*−) (equipotential boundary shown by the dashed orange line) causes the voltage and current contact points to exchange positions and increases the sensitivity to longitudinal resistance due to the width of the regions labeled source and drain.

In our devices, external voltage and current terminals are connected to the source and drain of 13 QHE elements, as shown in Fig. 1(a). NbTiN films form parallel superconducting interconnections to the 13 elements, with resistances *r*_*c*_ ≈ 1 Ω at the 25-nm-thick Pd/Au underlayer which forms the contact interface to the two-dimensional electron gas (2DEG) [see Fig. 1(b)]. Multiple connections to the equipotential edges are central to QHR arrays, as described by Delahaye [15] and investigated by Jeffery and co-workers [16]. Multiple contacts are a topological solution that allows precise two-terminal QHE measurements as if the device had ideal contacts (*r*_*c*_ = 0) for devices with longitudinal resistivity *ρ*_*xx*_ ≈ 0.

In Fig. 1(c), the superconductor wraps around from the source to the top Hall contact, and from the drain to the opposing Hall contact. For fixed total current *I*, most current flows into the 2DEG near the corners of the source-drain contacts (hot spots) as selected by the magnetic field vector. If the contact resistances *r*_*c*_ are too large, then some current could flow through the final Hall contacts; however, the error decreases by a factor *ϵ* ≈ (*ρ*_*xx*_ + *r*_*c*_)/*R*_K_ for each added edge connection to a quantized 2DEG element [15,16] provided that the metallic reservoir contacts are well separated [17].

Before all measurements, these devices were functionalized using chemical treatment to obtain *η*^6^-coordination of Cr(CO)_3_ to EG [18,19]. This forms equidistant bonds to three C atoms, preserving the planarity and superior electronic properties while providing a large drop in the carrier density of EG. This treatment provides *p*-type surface functionalization to counteract *n*-type doping of ≈1 × 10^13^ cm^−2^ induced in pristine EG on SiC(0001) by the substrate interface. Gentle heating at 40 °C–100 °C provides fine adjustment of the charge functionalization by removing weakly bound molecules adsorbed from the atmosphere.

## II. QUANTIZATION OF THE HALL RESISTANCE

We will present measurements at several carrier concentrations *n*_0_ produced by gentle heating in vacuum for device 1 and device 2, which share the same 4H-SiC(0001) substrate. In the parallel arrays, we cannot obtain *n*_0_ from the low-field slope of the Hall resistance due to the *R*_*xx*_ contribution at low *B*. At sufficiently high carrier densities, we can reliably derive *n*_0_ when the *ν* = 6 plateau near *R*_K_/78 ≈ 331 Ω is resolved as described in Sec. II D. We then use the slope from the origin to the approximate *ν* = 6 plateau center to find *n*_0_ and estimate the mobility *μ*.

### A. Initial characterization

**FIG. 2.**
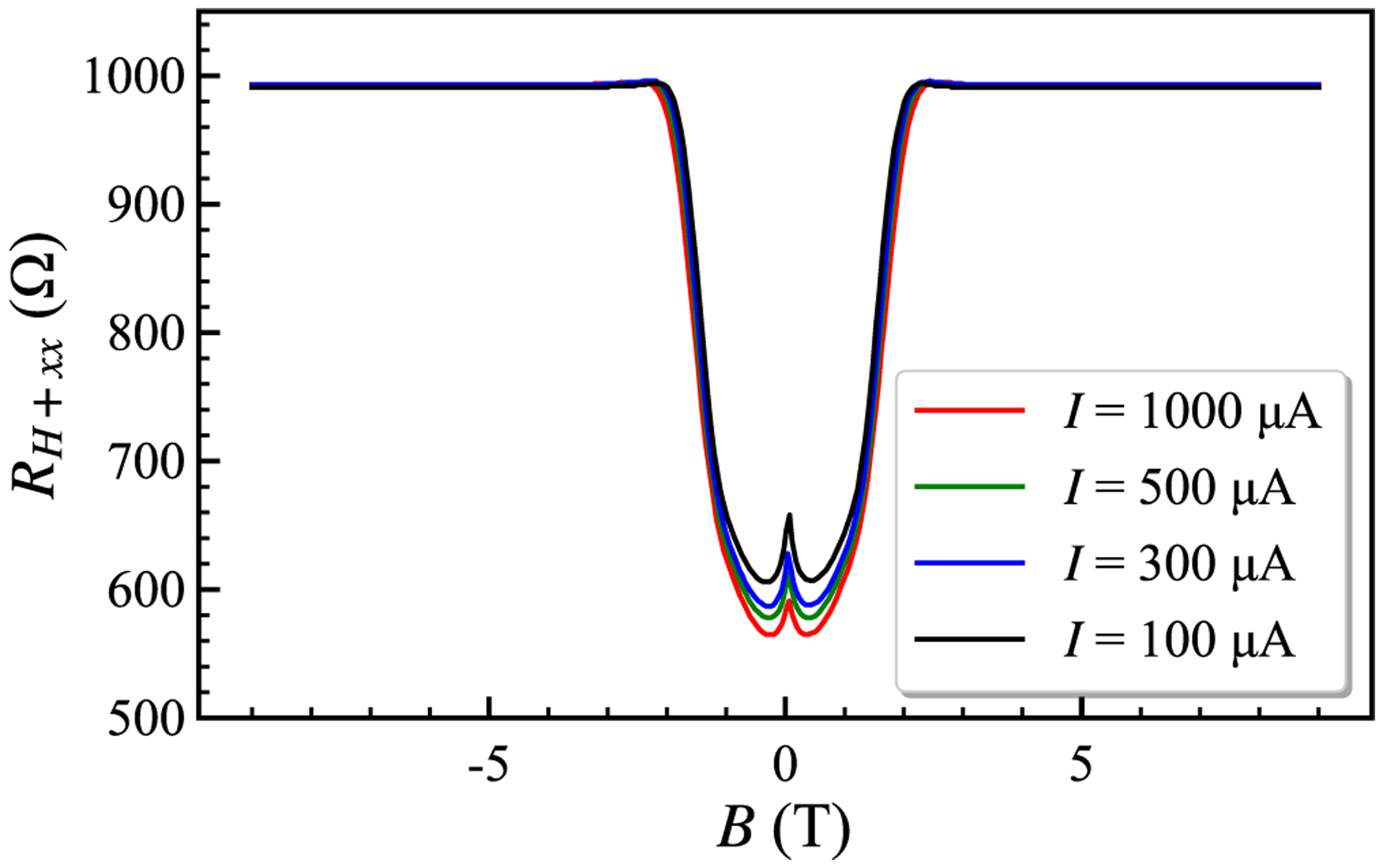
Resistance of array device 2 as a function of magnetic field B measured using a current source and a digital voltmeter. The symmetric resistance profile for *B*− and *B*+ typifies the combined voltage and current interconnections of a parallel array.

Figure 2 shows low resolution results for the array resistance at *T* = 1.6 K while sweeping the magnetic field. The symmetric form of the resistance profile is caused by reciprocity between voltage and current, as shown by the green and orange lines in Fig. 1(c). Onsager-Casimir reciprocity [20] with magnetic field reversal exists for linear electronic circuits and is well-known in QHE systems [6,15,17]. The observed magnetoresistance peaks centered at *B* = 0 are ascribed to weak localization resistance in monolayer graphene [21]. The decreased peak height implies electron heating in the high-current regime [22].

**FIG. 3.**
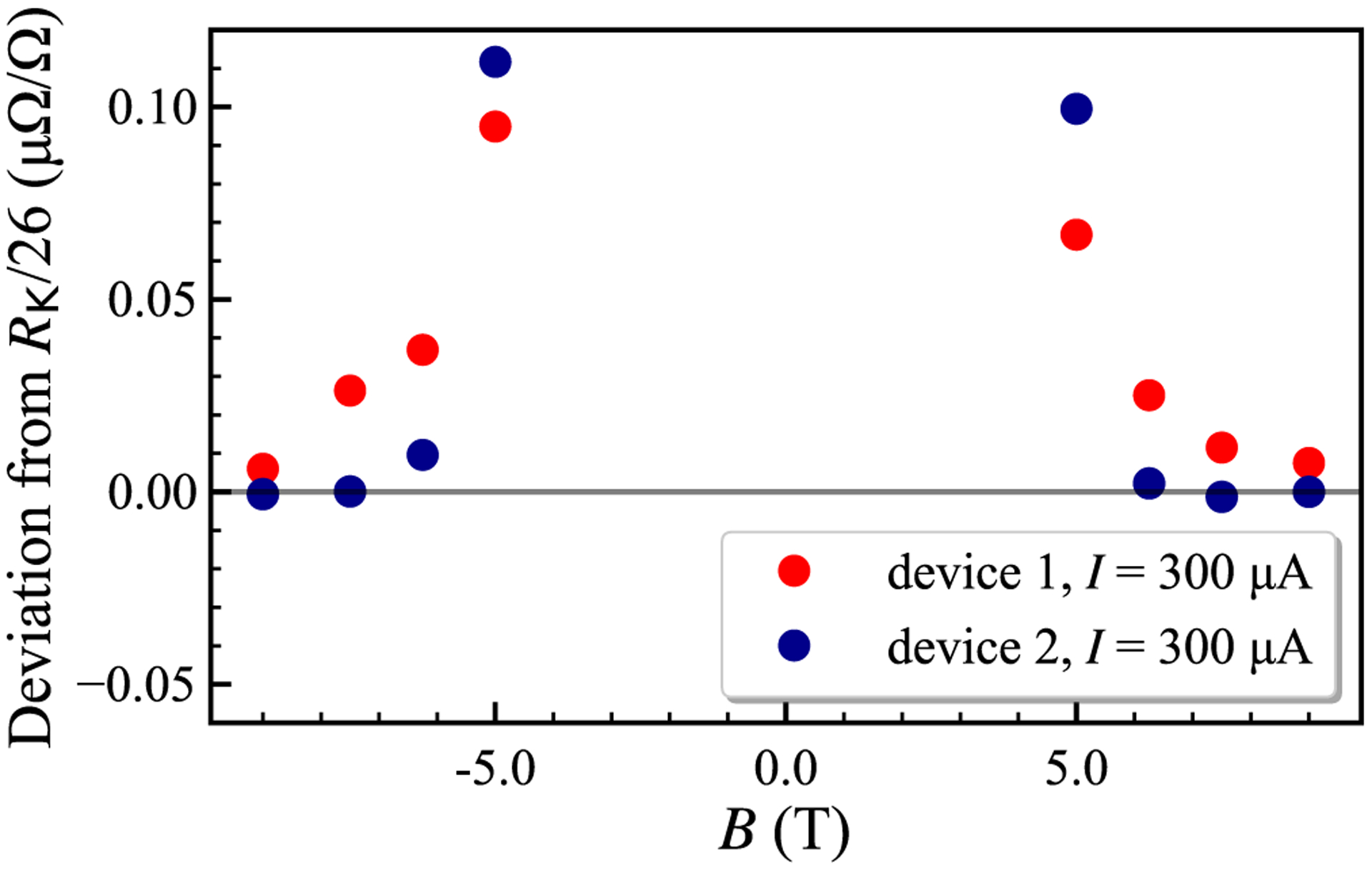
Symmetric cryogenic current comparator bridge data for two devices at 1.6 K, showing precise reciprocity with magnetic field reversal. Device 1 does not reach full quantization at *|B|* = 9 T. The results obtained on device 2 are quantized at 7.5 and 9 T, with deviation from the quantized value (−0.45 ± 4.47) n Ω/Ω when averaged for both field directions. Standard deviations are smaller than the size of the markers.

Cryogenic current comparator (CCC) bridge [23] measurements of two similar 13-element arrays are shown in Fig. 3. Device 1 is not perfectly quantized for *I* = 0.3 mA at *T* = 1.6 K, with relative deviation near 10^−8^ at ±9 T. Device 2 achieves full quantization above |7| T. For *B*+ and *B*− the deviations are (−0.65 ± 6.32) and (−0.25 ± 6.32) nΩ/Ω, respectively, at SI resistance value *R*_K_/26 ≈ 992.800 287 Ω. CCC ratio uncertainty is below 10^−9^, and most uncertainty originates from our 100 Ω artifact references. All expanded uncertainty values given here are for a 2*σ* confidence interval.

The room-temperature direct current comparator (DCC) bridge is more affordable than the CCC, requires much less training, and is widely used by calibration laboratories for low and moderate resistance values (*<*100 kΩ). DCC ratios are stable at the level of 10^−8^, and we regularly calibrate the DCC ratios using the same ratios of the CCC. Our choice of 13 QHR elements in parallel provides optimum DCC ratio sensitivity in automated bridge designs. All measurements that follow were made with a calibrated DCC, with the sample immersed in liquid ^3^He.

### B. Atmospheric doping

As described by Chuang *et al.* [24], we use atmospheric chemical doping to reversibly modify the electronic properties of graphene, including carrier density and mobility. The initial growth of graphene on hexagonal, basal plane SiC(0001) creates an interface layer of carbon with covalent bonds to some Si atoms on the SiC silicon-face surface [25,26]. The electronic configuration of the carbon atoms that bind to Si is *sp*^3^-hybridized rather than *sp*^2^ as in free-standing graphene [27]. The interface layer is nonconducting but the electronic *sp*^3^ hybridization induces *n*-type doping in the conducting epitaxial graphene layers that form later and lie above the interface layer [28,29]. Atmospheric molecules adsorbed on EG tend to act as electron acceptors, and thus reduce the *n*-type doping. This doping process involves several atmospheric gases with various rates of adsorption [19], as has been observed over periods of hours to months [30,31]. When Cr(CO)_3_ *η*^6^-graphene hexahapto functionalization is produced by reaction with heated Cr(CO)_6_ vapor, some of the atmospheric dopant molecules are replaced by Cr(CO)_3_, but many carbon atoms remain exposed to readsorb these dopants when exposed to ambient air. The Cr(CO)_3_ functionalization is stable for gentle heating up to about 150 °C, and atmospheric doping is reduced by heating at lower temperatures, thus heating in vacuum can be used to drive off atmospheric dopants and adjust the carrier density. Our results with Cr(CO)_3_ show that the carrier density can be adjusted from *p*-type to *n*-type, or from lower *n*-type to higher *n*-type levels by such heating, as reported in several prior publications [32–34].

### C. Device fabrication

**FIG. 4.**
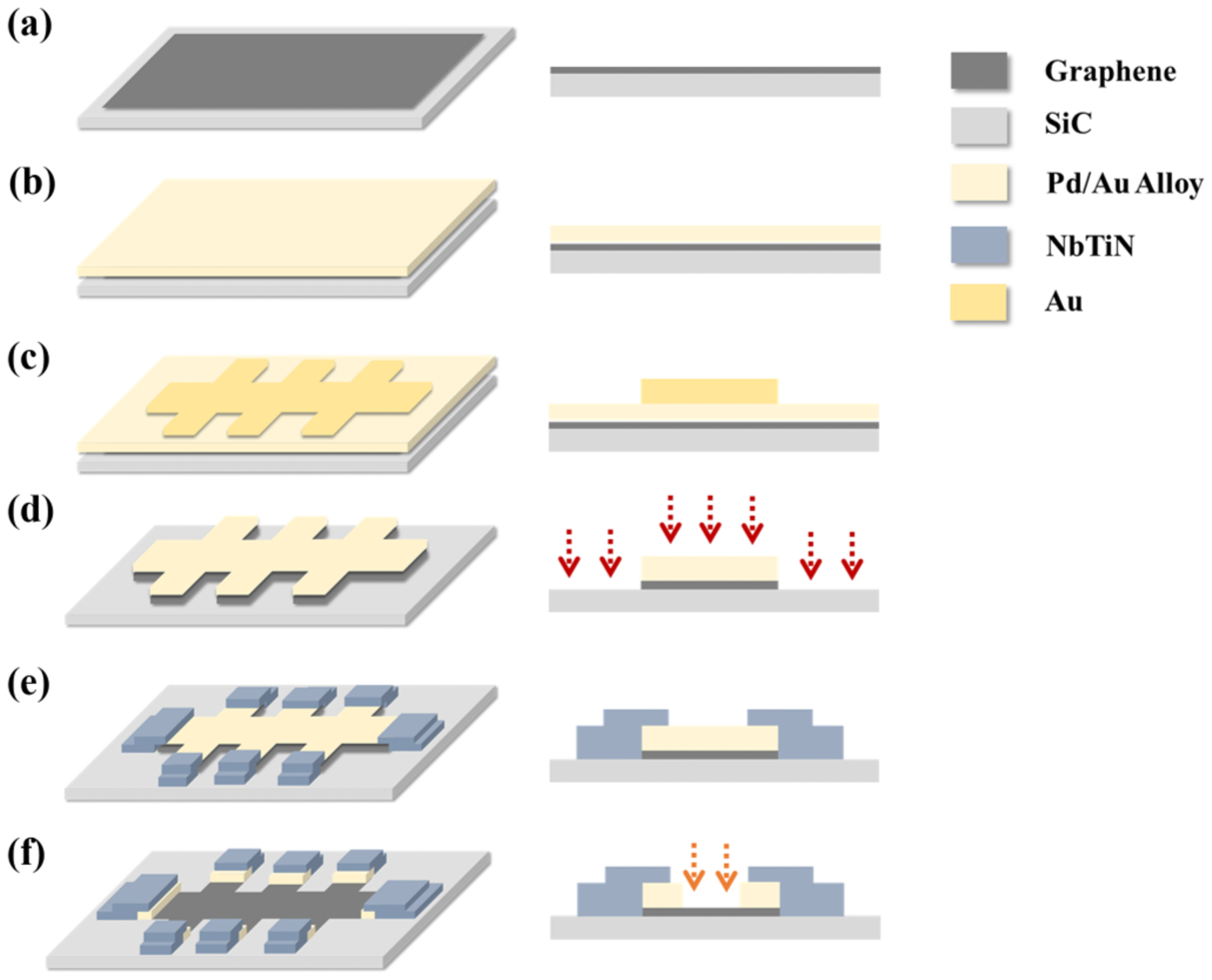
(a) As-grown graphene. (b) Protection layer deposition. (c) Sacrificed layer deposition. (d) Definition of the device pattern with ion milling. (e) Superconductor electrode deposition in a sputter chamber. (f) Removal of the sacrificial layer with dilute aqua regia.

Our lithographic fabrication process is shown in Fig. 4. First, a protection layer (10 nm Pd/15 nm Au) is deposited on the graphene with an e-beam evaporator. The protection layer is used to prevent the contamination of graphene during the fabrication process [34]. Pd is used here to uniformly cover graphene [35], and gold is used to prevent the oxidation of the Pd. After depositing the protection layer, we deposited 80 nm gold with a Hall bar pattern which acts as a sacrificial layer. The sacrificial layer is used as a metal mask for the following processing. Then, we use ion milling to define the EG regions on the surface of the device. Ar ions will uniformly etch the surface of the device so that only the regions covered with the sacrificed layer will remain after ion milling. The superconducting electrodes (NbTiN) are then sputtered on the device on top of the Pd/Au contact regions, and they do not directly touch graphene to prevent the interference between the quantum Hall state in graphene and the superconducting state in NbTiN [36,37]. Finally, we use dilute aqua regia to remove the protection layer on the graphene and then functionalize the graphene surface with Cr(CO)_3_.

### D. Carrier density tuning

**FIG. 5.**
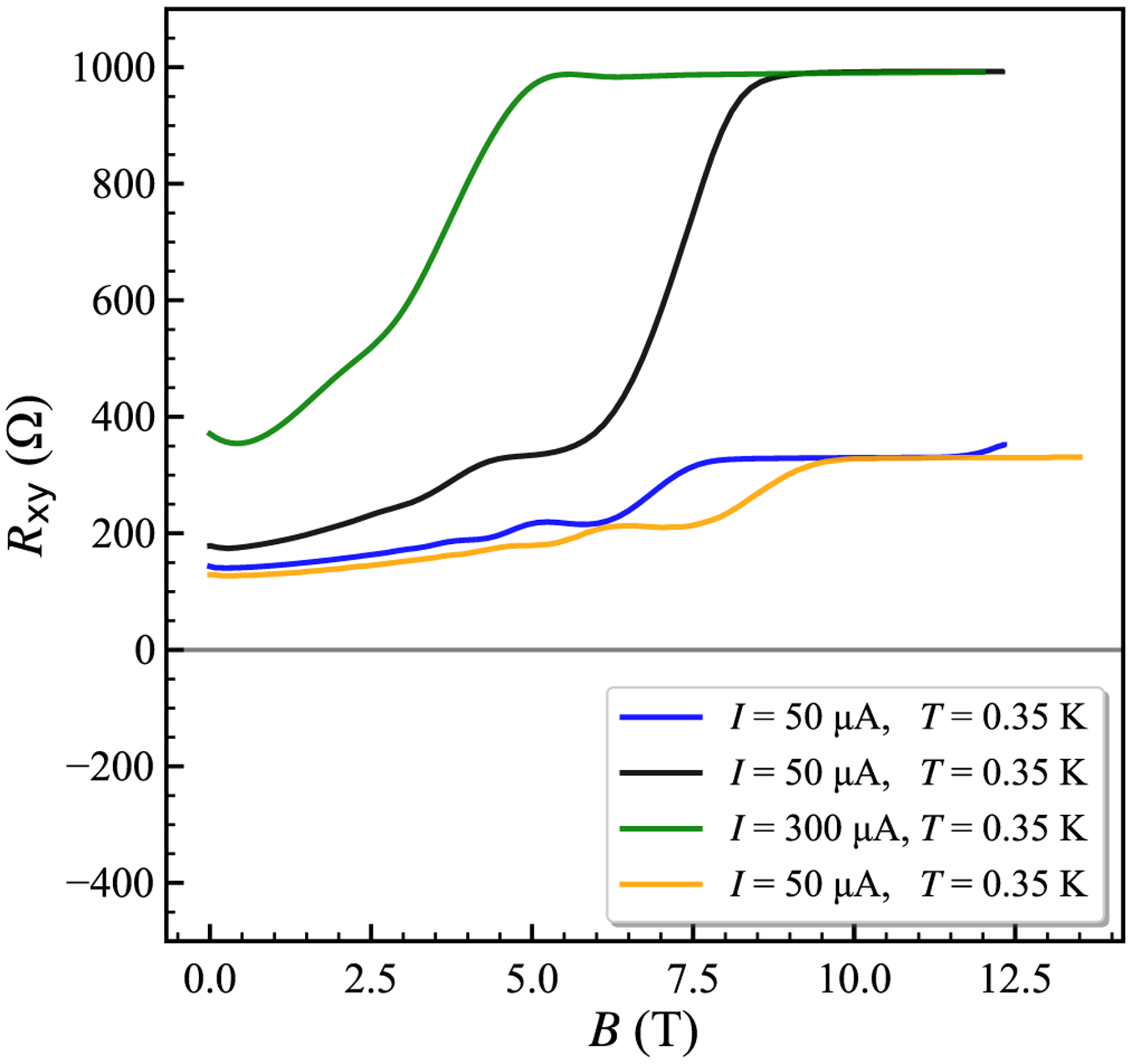
Measured QHARS resistance using a current source and digital voltmeter, for several carrier density levels (*n*_0_). Starting with the highest resistance at *B* = 0 (green), heating in vacuum was used to increase *n*_0_ to around 7 × 10^11^ cm^−2^ (black). Subsequent heating cycles in vacuum were used to produce the blue curve with *n*_0_ ≈ 1.6 × 10^12^ cm^−2^ (not discussed in the text), and the yellow curve with *n*_0_ ≈ 1.7 × 10^12^ cm^−2^, where a broad plateau is observed near 330.93 Ω.

**FIG. 6.**
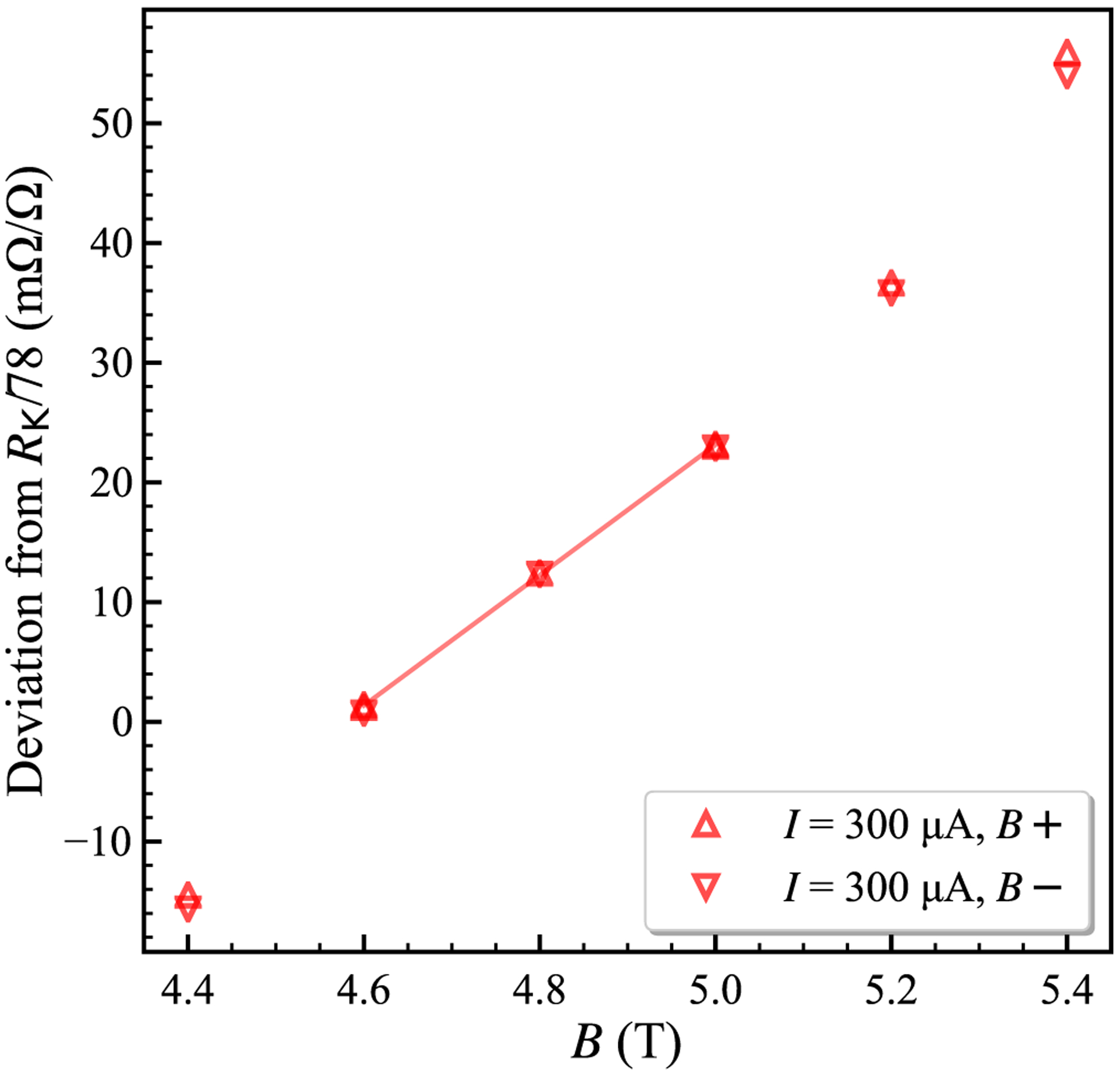
The region near 330.93 Ω for the black curve of Fig. 5, with *n*_0_ ≈ 7 × 10^11^ cm^−2^. The *ν* = 6 plateau is not visible, but these points show the minimum slope region measured using the DCC bridge at resistance levels near *R*_K_/78. The slope of the fitted line is ≈54.5 (mΩ/Ω)/T and the plateau center was estimated as *B* ≈ 4.8 T.

**FIG. 7.**
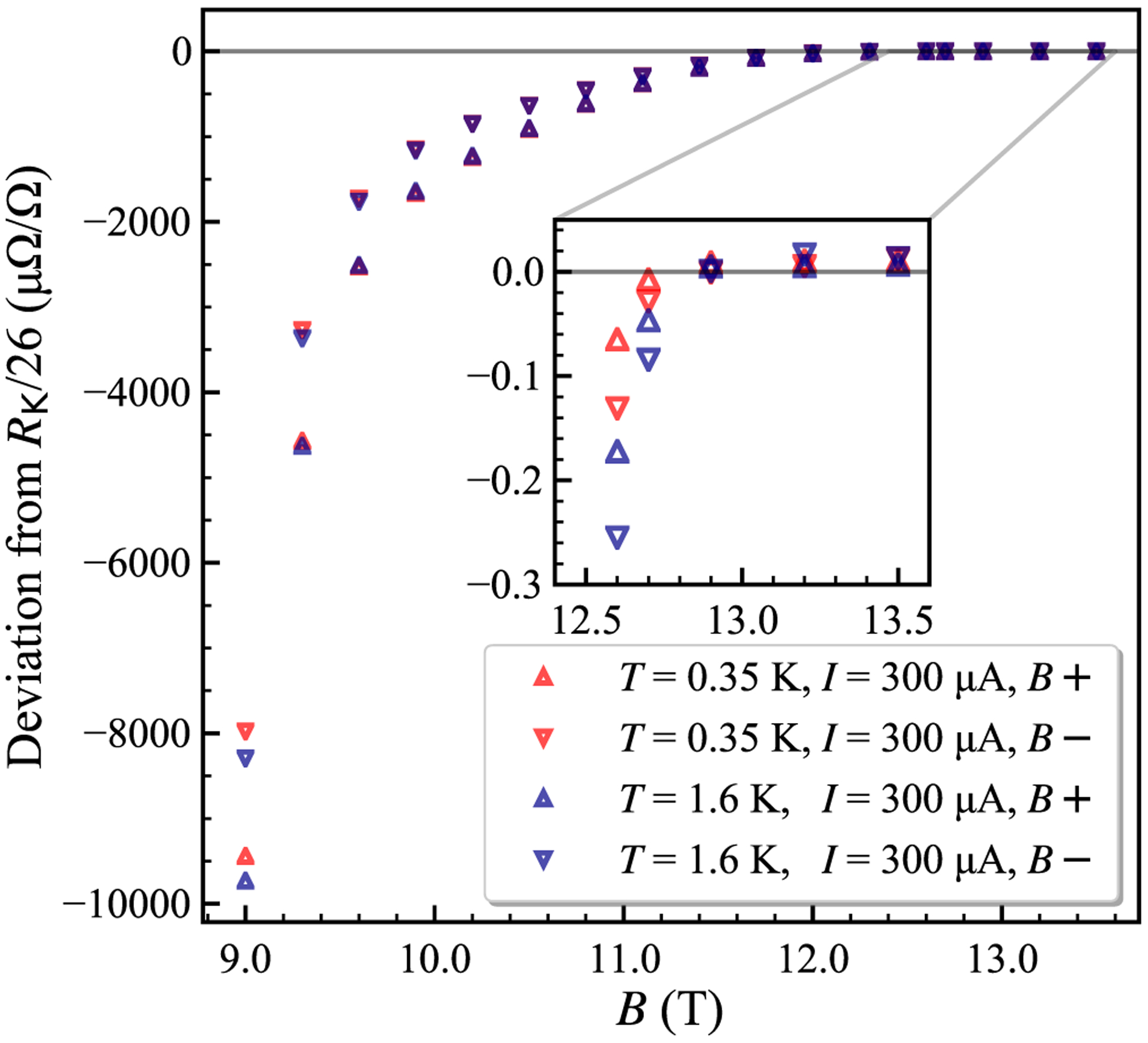
The *ν* = 2 plateau measured at selected values of *B* for device 1 at two temperatures (0.35 and 1.6 K) in a ^3^He cryostat using a room-temperature direct current comparator (DCC). The carrier density was determined as *n*_0_ ≈ 7 × 10^11^ cm^−2^ as shown by the black curve in Fig. 5. The inset shows data with a much finer scale, where the quantized plateau is seen to begin around 12.8 T for *B*+ and 13 T for *B*−. In general, the resistance values for *B*+ are closer to the quantized value for field values shown in the inset, but at somewhat lower fields the *B*− results are displaced closer to the plateau by increased longitudinal resistance.

We characterized the QHR arrays at the plateaus shown in Fig. 5, where both *ν* = 2 and 6 regions are present for some carrier densities. Heating in vacuum [24] was used to increase the *n*-type carrier density in our devices by ≈40%, with *n*_0_ ≈ 7 × 10^11^ cm^−2^ and *μ* ≈ 4000 cm^2^/V s for both device 1 and device 2, in agreement with values derived from the low-field Hall slope for large-area single EG devices produced by similar methods [38,39]. The broad magnetoresistance features are given by the black curve in Fig. 5, and precise DCC ratios were measured based on the same group of 100 Ω standard resistors (see Fig. 6). *B*+ yielded a *ν* = 2 resistance plateau value ≈(7.3 ± 6.9) × 10^−9^ higher than *R*_K_/26 at *T* = 0.35 K, starting from *B* = 12.9 T to maximum field *B* = 13.5 T (see Fig. 7). This offset in resistance quantization is consistent with earlier CCC data (Fig. 3, green curve in Fig. 5) at *T* = 1.6 K and *B* = 9 T, where carrier density *n*_0_ can now be estimated as 4 × 10^11^ cm^−2^ by comparing the *ν* = 2 plateau onsets.

**FIG. 8.**
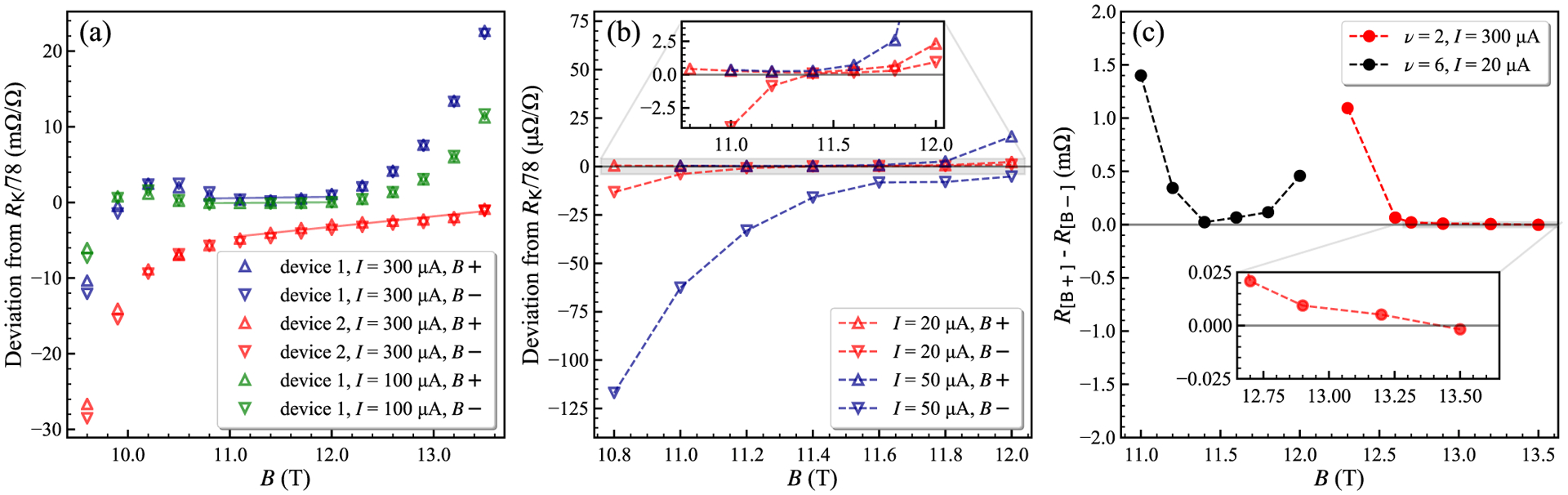
Resistance quantization measurements for device 1 at *T* ≈ 0.35 K. Standard deviations are smaller than the size of the markers, as derived from DCC bridge ratios. (a) The *B*+ and *B*− resistance for the *ν* = 6 plateau near *R*_K_/78 ≈ 330.933 429 Ω, where fitted lines are offset from the quantized value over a range of 1 T, with the scale in mΩ/Ω. (b) The *ν* = 6 resistance differs from the quantized value by (0.247 ± 0.054) *μ*Ω/Ω for *I* = 20 *μ*A and by (0.402 ± 0.027) *μ*Ω/Ω for *I* = 50 *μ*A over a range of 0.6 T in *B*+, from 11 to 11.6 T; the scale is in *μ*Ω/Ω. For the plateau center of 11.2–11.4 T, the measured deviation is (0.171 ± 0.076) *μ*Ω/Ω at 20 *μ*A and (0.264 ± 0.078) *μ*Ω/Ω at 50 *μ*A. The expanded uncertainties are for a 2*σ* confidence interval. (c) Comparing the magnetic field reciprocity at the *ν* = 2 and 6 filling factors.

**FIG. 9.**
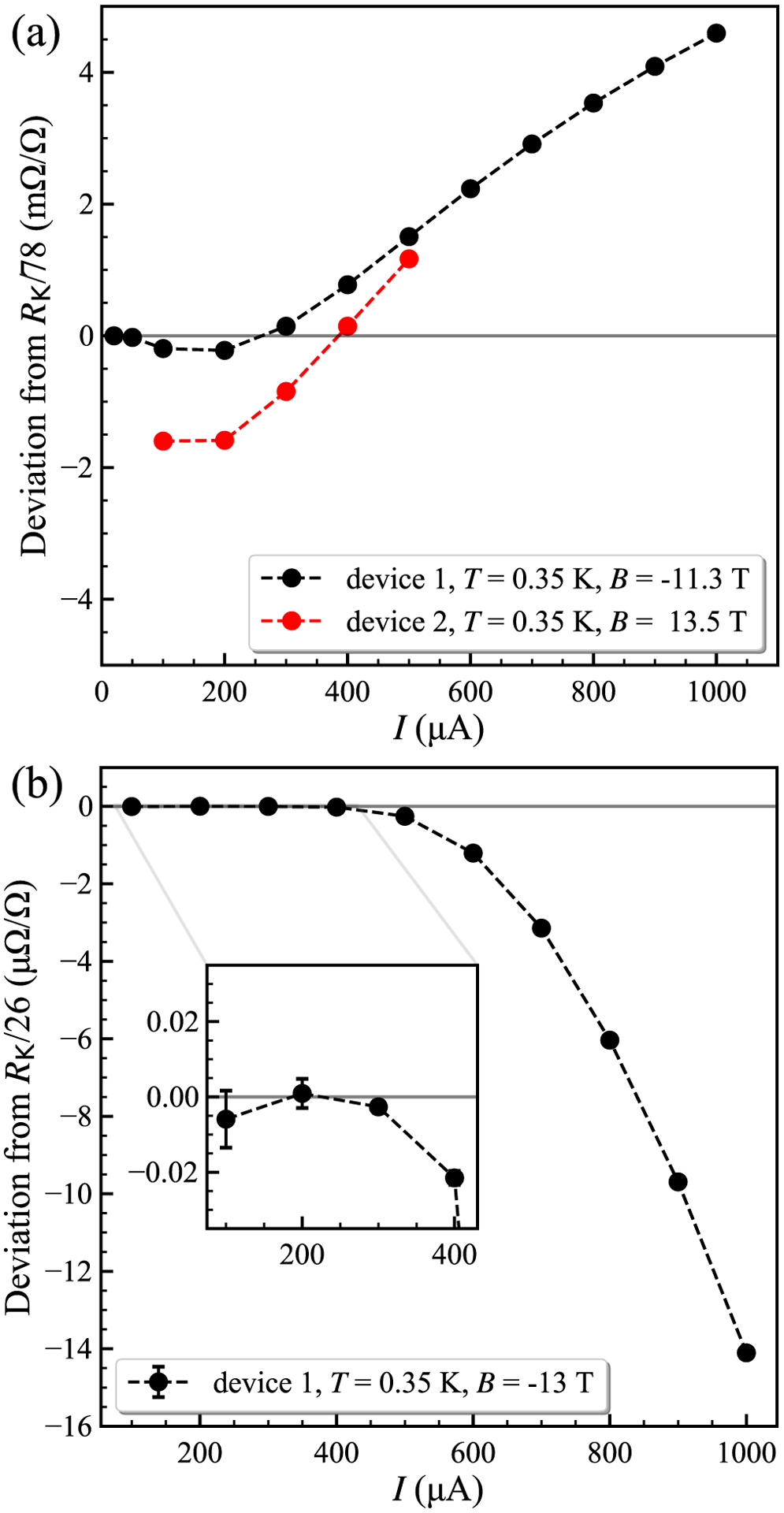
Current dependence. (a) The *ν* = 6 region, for device 1 and device 2, measured at two carrier density values. Quantization at the *ν* = 6 plateau was not attained for device 2, even at the higher carrier density. (b) The *ν* = 2 plateau resistance in device 1 measured at *B* = −13 T with source-drain current levels from 100 to 1000 *μ*A for the carrier density *n*_0_ ≈ 7 × 10^11^ cm^−2^. Inset: The values obtained at and below 300 *μ*A are consistent with full quantization at *R*_K_/26 with error bars showing the standard uncertainty of the DCC bridge data. The increase in the deviation at higher currents is proportional to the increase in power dissipation, or *I*^2^*R*.

We then heated the devices in vacuum again to obtain a much higher carrier density, yielding results shown in Figs. 8(a) and 8(b). Here *n*_0_ ≈ 1.7 × 10^12^ cm^−2^, and we estimate *μ* ≈ 2300 cm^2^/V s. The *ν* = 6 plateau, now centered near 11.4 T, was investigated using both devices with the DCC bridge for *I* = 300, 100, 50, and 20 *μ*A (see the orange curve in Fig. 5). Device 1 displayed an approach to quantization near *R*_K_/78, however we see a strong dependence on the device current, much stronger than expected from the current dependence for the *ν* = 2 plateau (see Fig. 9). The current dependence is reduced for the low current range between 50 and 20 *μ*A, and a reasonably flat and broad plateau appears for *B*+ at 20 *μ*A as shown in Fig. 8(b). The deviation of the central plateau at *ν* = 6 for 20 *μ*A is (0.17 ± 0.08) *μ*Ω/Ω. The approach to reciprocity at the plateau centers is demonstrated in our devices at the two carrier densities where the *ν* = 6 and 2 plateaus in Fig. 8(c) begin at similar ranges of *B*, although the reciprocity range where *R*(*B*+) − *R*(*B*−) ≈ 0 is small for the *ν* = 6 plateau.

## III. DISCUSSION

### A. Metrological requirements of quantized Hall array resistance standards

GaAs/AlGaAs heterostructure-based quantized Hall array resistance standards have demonstrated relative precision of a few parts in 10^9^, approaching that of single devices, both at large and small values relative to *R*_K_ [5–7]. The device elements (typically of quantized resistance value *R*_K_/2) in a parallel array with *N* elements share the current nearly equally allowing an increase in the measurement sensitivity with higher total current. Joule heat is dissipated in two small areas of each element adjacent to the contact points where current enters and exits the two-dimensional electron gas (2DEG). For small relative resistance differences Δ_*n*_ (*n* = 1, 2, … , *N*), the *N*-element array resistance is given by Taylor expansion as

(1) $$R=\frac{R_{\text{K}}}{2N}\left[1+\frac{\Delta_{1}}{N}+\cdots +\frac{\Delta_{N}}{N}\right].$$

When the ideal quantized plateau state is obtained for the QHE elements, all Δ_*n*_ should be too small to be measurable. In single QHR devices, one can follow international guidelines [40] to verify the ideal behavior of the QHR standard. Specifically, precise four-terminal measurements can verify that longitudinal resistance *R*_*xx*_ is negligible in the region of measurement, and three-terminal measurements can show that the resistance in the contacts is small compared to *R*_K_. These tests are impossible for arrays, because resistance measurements at the individual Hall contacts are inaccessible due to the permanent array interconnections between them.

As described earlier, arrays studied here have superconducting, crossover-free interconnections, and split contact design [37]. These new techniques successfully eliminate the accumulation of internal resistances and leakage currents that typically occur at interconnections and lead crossings between interconnected devices. The high critical magnetic field of the NbTiN superconductor ensures superconductivity up to *T* ≈ 6 K for magnetic flux density of 13.5 T. At *B* = 0, the typical critical transition temperature is 12.5 K [37]. Thus, in the ideal QHE condition where *ρ*_*xx*_ ≈ 0 for all elements, the EG array closely approximates a condition where the chemical potentials at the 2DEG Hall terminals are equal to the respective potentials of the superconducting source and drain.

Empirical studies show that the longitudinal resistivity, *ρ*_*xx*_, in the 2DEG of GaAs-based heterostructures for fixed magnetic flux contributes to the deviation in Hall resistivity, Δ*ρ*_*xy*_, as [41]

(2) $$\Delta \rho_{xy}\approx s\rho_{xx}.$$

Here, *s* represents the effect of geometrical contact misalignment and microscopic 2DEG disorder. Equation (2) was shown to be applicable over three or four orders of magnitude in *ρ*_*xx*_ for conditions of variable temperature and power dissipation close to the plateau center in the QHE regime.

### B. Landau-level broadening

Landau-level quantization in monolayer graphene is fourfold-degenerate; at moderate magnetic fields, only the quantum occupation numbers *ν* = (2, 6, 10, … .) appear in a sweep of *R*(*B*), and only the *ν* = 2 plateau is fully quantized. To our knowledge, no metrological measurements have been attempted previously with graphene in the *ν* = 6 quantized state, although both the *ν* = 4 and 2 plateaus were often used for metrology of GaAs/AlGaAs heterostructure-based devices in the 1980s and 1990s [41]. In semiconductor-based 2DEGs, the separation of the LLs is linear with the magnetic flux density *B*. In contrast, the energies of the LL states in graphene are given by

(3) $$E_{n}=\text{sgn}(n){\left[2e\hbar v_{\text{F}}|n|B\right]}^{1/2}+E_{0},\;n=0,\pm 1,\pm 2,\ldots .$$

Here *e* is the elementary charge, *ħ* is the reduced Planck constant, *ν*_F_ is the Fermi velocity, *n* is the LL index, and *E*_0_ is the Dirac point energy. Thus, the LL center energy separation (*E*_2_–*E*_1_) above *n* = 1 is about 0.41 times as wide compared to the separation above the *n* = 0 LL, which is occupied equally by both electrons and holes. However, the degree of quantization is determined by the broadening of the LLs as well as their spacing.

Theoretical and experimental reports have raised questions concerning the broadening of LLs in monolayer graphene. Yang, Peeters, and Wu [42] included electron-impurity (e–i) and electron-electron (e-e) interactions, LL coupling, Fermi energy, and magnetic-field-dependent contributions. Funk, Knorr, Wendler, and Malic [43] found that the LL broadening is mainly due to e-i interactions and proportional to *B*^1/2^, counteracting the similar dependence on separation between graphene LLs. They note experimental evidence by Orlita *et al.* [44] indicating that the number of resolvable LL optical transitions is constant for a range of magnetic field strengths in EG, suggesting a dependence on (*nB*)^1/2^ in the LL broadening that cancels the increased LL spacing. For conventional 2DEG systems, the e–i broadening also scales as *B*^1/2^, but the spacing of the LLs increases linearly with *B*, increasing the separation and reducing the overlap in the density of states. Our results extend the study of LL broadening in EG, although we do see evidence from the plateau slope in Figs. 5, 8(a), and 8(b) that the LL overlap at *ν* = 6 decreases for higher *n*_0_ and produces a stronger plateau at higher *B* in the same device.

The deviation from quantization and the plateau evolution for the observed filling factors in electrical measurements is dependent on carrier density (in part due to e–e interactions and screening effects), mobility (e–i interactions and screening), temperature and source-drain current (phonons and thermal activation), and other factors. Up to now, detailed experimental studies of the interplateau transitions by varying these parameters have not been possible at a high level of precision.

Modifying the carrier density in EG causes a reciprocal change in the mobility, as shown by several studies [38,45]. It is likely that the *ν* = 6 plateau quantization may be degraded by the reduction in mobility that occurs along with the higher carrier density needed to observe this plateau.

It is well known that the presence of impurities broadens and obscures fractional QHE states [46–48] that lie close in energy, but also that some disorder is needed to produce broad LL plateaus for the integer QHE.

The scaling properties of condensed matter systems are of great interest, and such effects have been the basis of much theoretical and experimental work [49,50]. We note that most theoretical studies of LL broadening are on graphene on SiO_2_. Most studies of EG have focused on the *ν* = 2 quantum Hall plateau, which extends to high *B* due to charge transfer from the SiC substrate, and has also been observed in graphene covered by InSe [14,51]. Therefore, additional experimental and theoretical work focused on LL broadening specifically for graphene on SiC will be highly desirable in the search for improved and accessible electronic standards as well as a better understanding of the underlying interactions.

## IV. CONCLUSION

In summary, we have described the QHR realized in EG devices constructed with 13 QHE elements in parallel, measured at the two plateaus that lie between the zeroth and second LLs. Carrier density adjustment is demonstrated using simple low-vacuum techniques. The carrier density is quite uniform over large sample areas of 1 mm × 5 mm, and the density of impurities that scatter electrons is also uniform for these EG devices, as shown by the width of the *ν* = 6 plateau at higher carrier densities and mobility of order *μ* ≈ 4000 cm^2^/V s at relatively high carrier density *n*_0_ ≈ 7 × 10^11^ cm^−2^.

These are the first graphene QHR arrays suitable as primary SI references, at a useful resistance level near 1 kΩ where room-temperature DCC bridges are most precise. The arrays have quantized resistance *R*_K_/26 with *ν* = 2 filling factor in each element. Quantized results for *ν* = 6 at resistance ≈*R*_K_/78 are less precise, but the measured offset of device 1 is approximately 2 × 10^−7^ near the plateau center for *I* = 20 *μ*A. Magnetic field reciprocity gives evidence on the longitudinal resistivity in our QHE arrays, but it has not been carefully studied in epitaxial graphene. Future work on interplateau transitions and reciprocity, using similarly fabricated QHE single devices as well as arrays, may provide a better understanding of theoretical and experimental aspects of the QHE.
